# First dispersal records of the endangered banteng (*Bos javanicus*) in Thung Yai Naresuan West Wildlife Sanctuary, Thailand

**DOI:** 10.1002/ece3.11602

**Published:** 2024-06-21

**Authors:** Peerawit Amorntiyangkul, Pornkamol Jornburom, Anak Pattanavibool, Warong Suksavate, Supalerk Klanprasert, Sahasawat Kaewvisat, Thanadid Thongthai

**Affiliations:** ^1^ Wildlife Conservation Society Thailand Program Nonthaburi Thailand; ^2^ Kasetsart University Faculty of Forestry Bangkok Thailand; ^3^ Royal Thai Government Department of National Park Wildlife and Plant Conservation Bangkok Thailand

**Keywords:** camera trap, dispersal, endangered species, large ungulate, protected area

## Abstract

Banteng (*Bos javanicus*) is listed as an endangered species because of a global population decline of at least 50% over the last 25 years. The Western Forest Complex (WEFCOM) of Thailand has been identified as a priority site for banteng population recovery, and Huai Kha Keang Wildlife Sanctuary (HKK) is the most important source site for this species within the WEFCOM. We have provided evidence and discussed banteng dispersal from HKK to Thung Yai Naresuan West Wildlife Sanctuary (TYW). We sampled an area of 147 km^2^ in banteng habitat next to the border between HKK and TYW using camera traps. We divided the sampled area into four grid cells and placed camera traps during January to December 2022. We setup the camera traps near saltlicks and natural water sources, as important resources for banteng, to maximize capture probability. In total, 2835 trap days were obtained. Bantengs were captured in all seasons (RAI = 1.66), especially in dry dipterocarp forest, which contains the ground forage availability for banteng, and the low‐slope area with elevation 600–700 m adjacent to the border between HKK and TYW. The results highlighted that banteng, which had never been reported in TYW before, appeared there for the first time. They most likely dispersed from the population source in HKK and settled in a habitat that is considered suitable for them. The habitat management and protection are significant for the future recovery of banteng populations in the TYW and the rest of protected areas in the WEFCOM.

## Graphical abstract

This article provided evidence and discussed the first dispersal of endangered banteng from Huai Kha Keang Wildlife Sanctuary, the core area of the world natural heritage site in Thailand, to Thung Yai Naresuan West Wildlife Sanctuary where the species has never been reported.

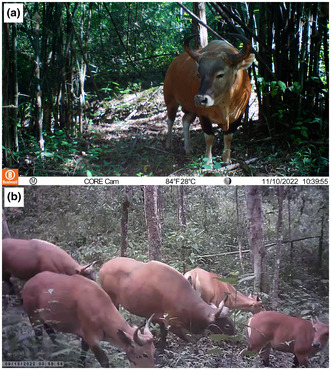

## 1 INTRODUCTION

The banteng (*Bos javanicus*) is a large herbivore belonging to the Bovidae Family with a natural geographical range limited to Southeast Asia (Gardner et al., 2016; Kongsurakan et al., 2020; Pedrono et al., 2009). It is listed as endangered with a recent population decline of at least 50%, currently existing in small isolated populations. In Thailand, until recently, its range and population were drastically reduced by approximately 80% (Gardner et al., 2016; WWF et al., 2023). Habitat loss and poaching for trophies and meat have been the key threats to the species (Gardner et al., 2016; Srikosamatara & Suteethorn, 1995). The Western Forest Complex of Thailand (WEFCOM) has the largest remaining population.

Large‐scale occupancy surveys conducted throughout WEFCOM during 2010–2012 indicated that banteng were only recorded in Huai Kha Khaeng Wildlife Sanctuary (Jornburom et al., 2020). HKK is the core area of the WEFCOM and an important area for banteng conservation. Expert opinion believed that HKK contained the population of 400–500 individuals (Chaichanathong et al., 2021; Phoonjampa et al., 2021). However, Saisamorn et al. (2024), with rigorous sampling methodology, have reported that banteng population in HKK has recovered to almost 3000 individuals due mainly good protection from poaching in the last 15 years. Jornburom et al. (2020) attempted to predict the potential distribution of banteng in WEFCOM and suggested that if poaching was effectively reduced, banteng can disperse from HKK into the Thung Yai Naresuan West Wildlife Sanctuary (TYW). Historically, the TYW has been disturbed by intensive human activities, for example, villages within the area, poaching from illegal gangs, site preparation for dam building, and mineral extraction that was suspended between 1981 and 1990 (Amorntiyangkul et al., 2022). Through the high quality of connectivity between HKK and other protected areas in WEFCOM, bantengs have a chance to disperse into other protected areas in WEFCOM, if population recovery happens in HKK (Phoonjampa et al., 2021; Prayurasiddhi, 1997; WWF et al., 2023). Following such predictions, we now report on the recovery of bantengs by showing, for the first time since wildlife distribution data have been recorded in Thailand, the concrete evidence of its dispersal from HKK to TYW.

## 2 MATERIALS AND METHODS

### 2.1 Study area

Thung Yai West Wildlife Sanctuary (TYW) is part of the Western Forest Complex (WEFCOM) and contiguous with Huai Kha Khaeng Wildlife Sanctuary (HKK). TYW, together with HKK and the Thung Yai East Wildlife Sanctuary, was inscribed as a UNESCO World Heritage Site since 1991 (Kanchanasaka, 1997; Saisamorn et al., 2019). We conducted a camera‐trapping survey in the southeastern section of TYW, where it is connected to HKK. We surveyed a valley and hill slope with elevations ranging from 600 to 1200 m. The forest cover includes dry evergreen, dry dipterocarp, and savannah grasslands (Amorntiyangkul et al., 2022; Duangchatrasiri et al., 2019). The average annual rainfall is 1900 mm and the temperature ranges from 7 to 37°C. The climate characteristics were classified into two seasons, wet (May to October), and dry (November to April) (Kanchanasaka, 1997).

### 2.2 Camera‐trapping survey

**FIGURE 1:**
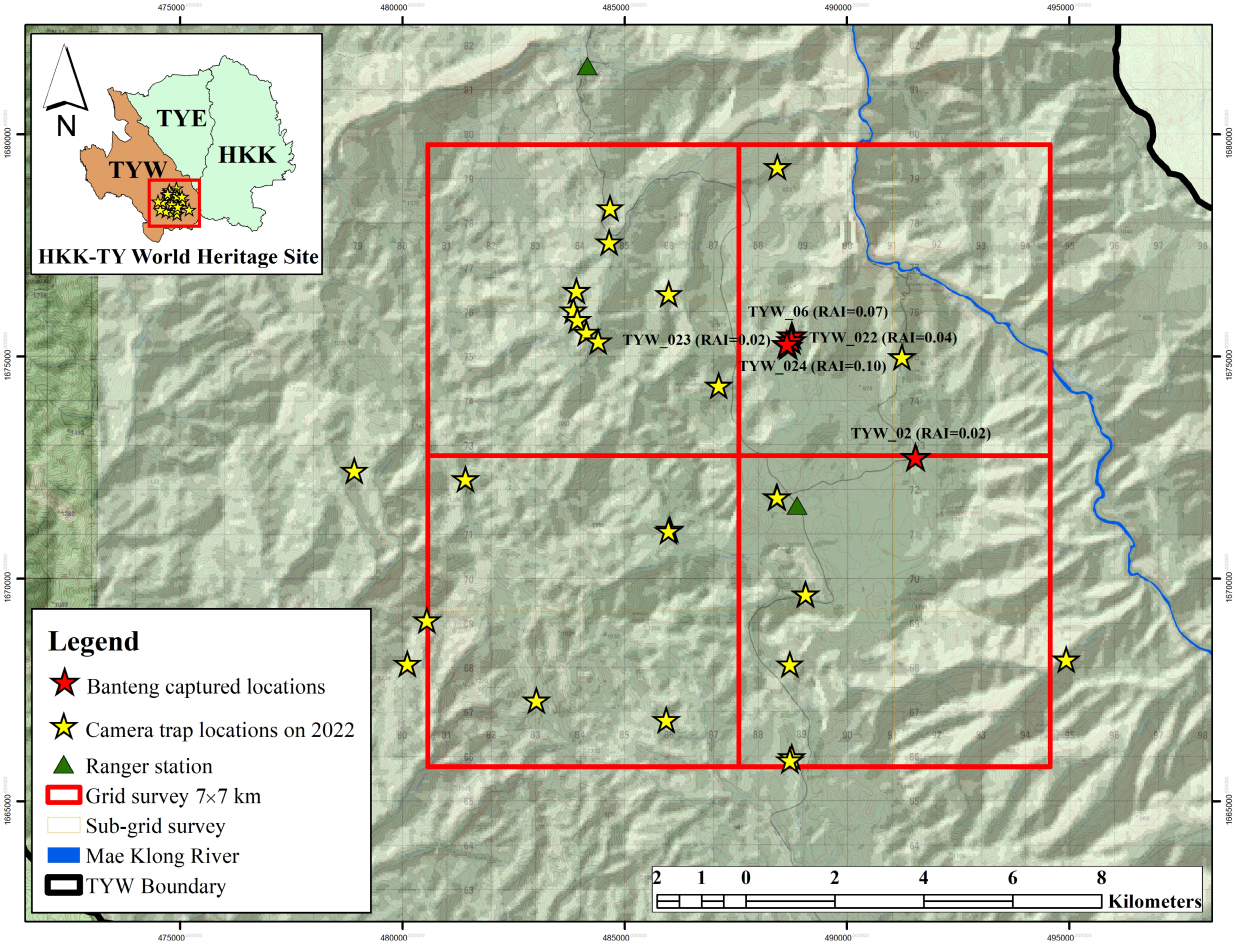
Map showing the study area in the Thung Yai West Wildlife Sanctuary (TYW), the area contiguous with Huai Kha Khaeng Wildlife Sanctuary (HKK), and the camera trap location setup from January to December 2022, covering 147 km^2^ on hill slopes between 600 and 1200 m a.s.l. The banteng locations are indicated in red.

A camera‐trapping survey was conducted to study banteng habitat use in TYW from January to December 2022. We sampled 147 km^2^ surrounding the Thikong Forest Ranger Station and divided the area into four grid cells of 7 × 7 km per grid cell, following the banteng home range of 49 km^2^ in Thailand (Prayurasiddhi, 1997) (Figure 1). Each large grid cell was further divided into four 3.5 × 3.5 km grid cells for camera trap spacing and placement to investigate banteng habitat use. For camera trap placement, we took into account the factors influencing banteng distribution and intensity of habitat use, especially near saltlicks and water sources, in order to maximizing capture probability (Suksavate et al., 2022; Thapa et al., 2019). We set camera traps at 33 locations using a combination of the camera trap models Bushnell DS‐4K and Spartan E4GB2, which were the main camera traps used in this study (Figure 1). Camera traps were placed 40–60 cm above the ground and set up along ridges, saltlicks, water sources, and wildlife trails (Silver et al., 2004; Suksavate et al., 2022). For data interpretation and analysis, we considered consecutive photographs with a time interval of more than 30 min as new photographic events (O'Brien et al., 2003; Rahman, 2019; Saisamorn et al., 2019). Banteng age class classification was defined for individuals in each capture event as adult (>3‐years old), juvenile (1–3 year‐olds), or calf (<1 years old), based on the body shape and size (Phoonjampa et al., 2021).

## 3 RESULTS

### 3.1 Banteng trap success in TYW

**FIGURE 2:**
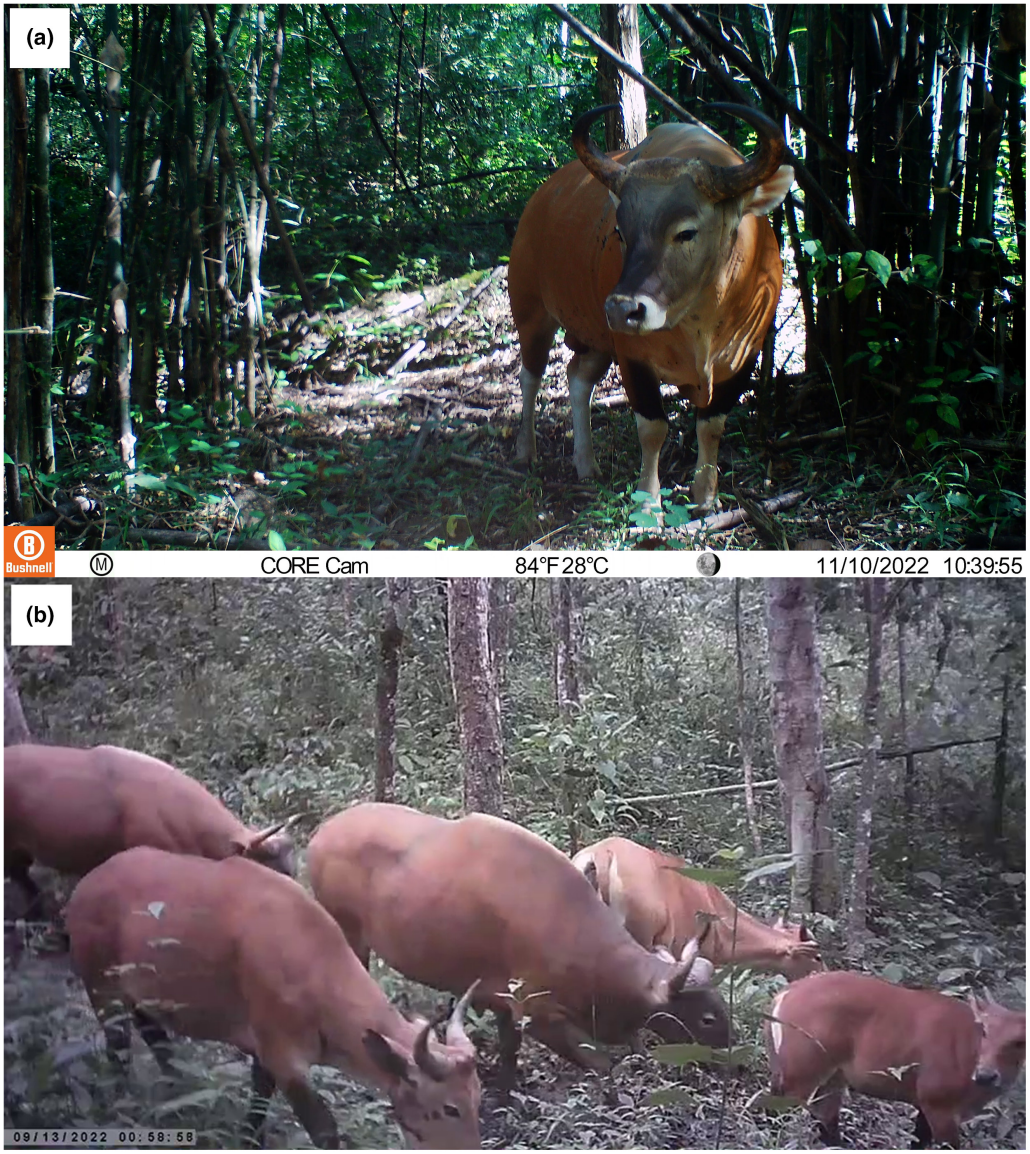
Photographs of banteng captured during the camera trap survey in Thung Yai Naresuan West Wildlife Sanctuary, showing the breeding potential of the banteng population. (a) Adult male and (b) a five‐banteng herd, including a calf.

Our camera trap survey in 2022 recorded the dispersal of bantengs from HKK to TYW for the first time. After 2835 trap days, we recorded bantengs at five locations during 47 independent events. We also captured banteng calves, indicating a breeding population in TYW (Figure 2). The largest herd of banteng contains six individuals, which captured during walking on animal trial to natural water source under the camera ID include TYW_06, TYW_22, TYW_23, and TYW_24 (Table 1). The banteng trapping rate per 100 trap nights was 1.66 (Table 1). They were captured in both the dry and wet seasons, especially around the low‐slope area near the eastern border between the HKK and TYW, with elevations ranging between 600 and 700 m. (Figure 1). The tiger (*Panthera tigris*) and other main tiger prey detected during the same period included wild boar (*Sus scrofa*), red muntjac (*Muntiacus muntjak*), sambar (*Rusa unicolor*), and gaur (*Bos gaurus*) (Jornburom et al., 2020).

**TABLE 1:** Camera trap effort and captured statistics at the five locations where banteng was detected in Thung Yai Naresuan West Wildlife Sanctuary from January to December 2022.

Camera locations	Season	Trap nights	Number of individual				Independent photographs	RAI ratio
			Adult	Juvenile	Calf	Sum		
TYW_02	Dry	86	1	0	0	1	2	0.02
TYW_06	Dry	183	3	1	2	5	13	0.07
TYW_22	Dry, Wet	206	4	1	0	5	9	0.04
TYW_23	Dry, Wet	232	3	0	1	4	4	0.02
TYW_24	Dry, Wet	183	5	2	2	9	19	0.10

## 4 DISCUSSION

**FIGURE 3:**
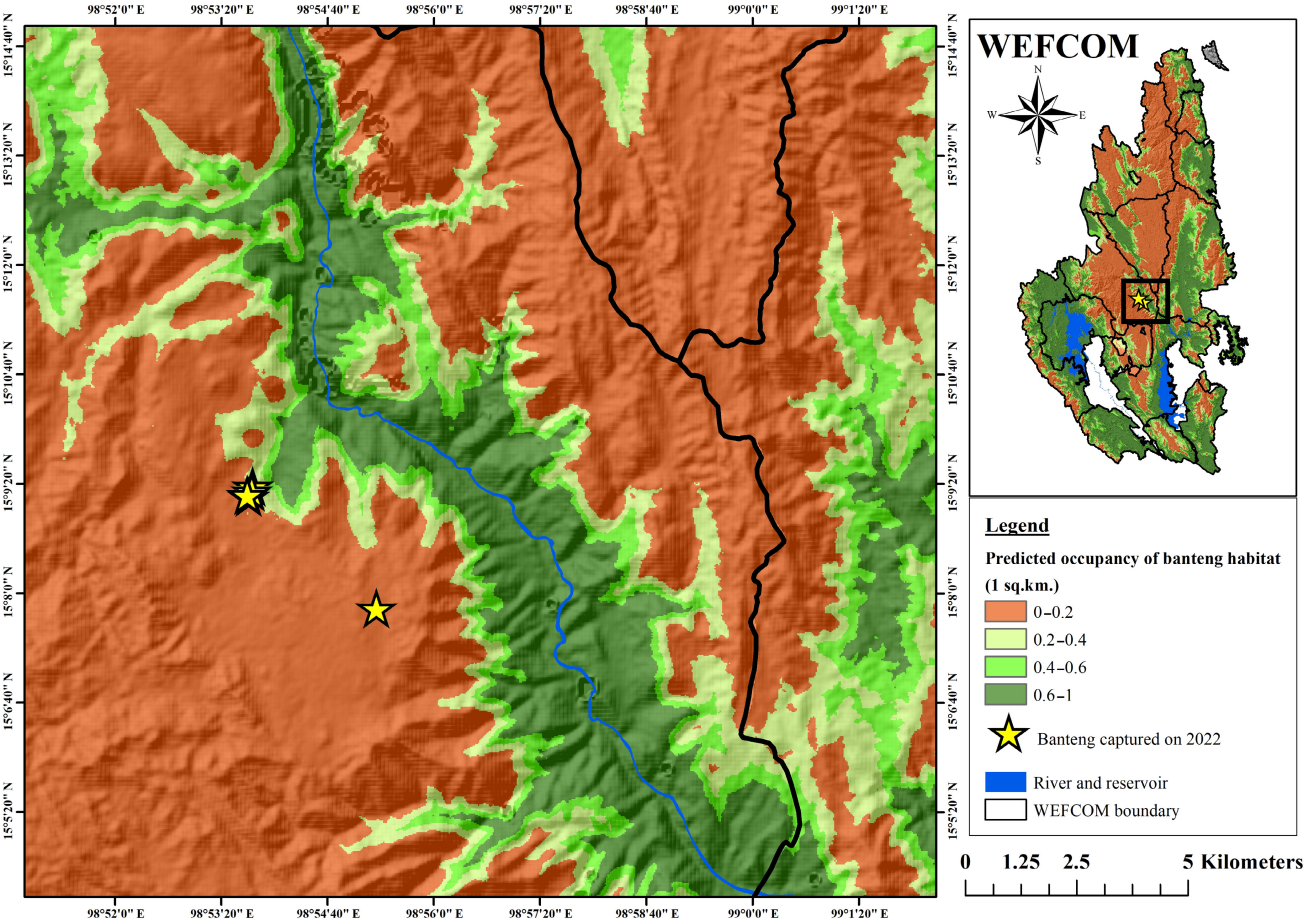
Map shows banteng locations (yellow stars) in the eastern section of Thung Yai West from the camera trap survey, with the potential distribution of banteng proposed by Jornburom et al. (2020) as background.

Our results highlight, for the first time, evidence of banteng in TYW that most likely dispersed from the source population in HKK. This is beyond the previous range reported by Prayurasiddhi (1997). It is coincide with the prediction by Jornburom et al. (2020) that, if poaching and other human distance in the area is controlled, some habitat in other protected areas next to HKK can support bantengs (Figure 3). In recent years, bantengs in HKK has significantly density increased from 0.83 in 2007 to 1.71 in 2022 (Saisamorn et al., 2024). The dispersal of bantengs from HKK into Mae Wong National Park, next to the north of HKK, was the first evidence of bantengs dispersing out of HKK (Phoonjampa et al., 2021). Resource availability and habitat connectivity are the key elements of dispersals of bantengs from HKK into neighboring protected areas. Ungulates can disperse through the rugged terrains into the new area with high forage availability (Killeen et al., 2014). In TYW, we detected bantengs in the undulating terrain and lower elevation (600–700 m), where saltlick and natural water source are abundant. Saltlicks are the important mineral source for wildlife, especially herbivores (Razali et al., 2020). Natural surface water is also important for wildlife species (Morgart et al., 2005). This area is covered by both dry dipterocarp forest and savannah grassland, suitable for bantengs. Although bantengs prefer dry dipterocarp forest (Steinmetz, 2004), they also use deciduous or evergreen forests with grassy glades that burn annually (Gardner et al., 2016).

Site security from human disturbance is also an important factor in areas with history of human disturbance (Suksavate et al., 2019; Vinitpornsawan, 2013). The distributions of ungulates were negatively responding to distance from villages in the WEFCOM with heavy human activities include poaching, bamboo cutting, and cattle grazing (Duangchatrasiri et al., 2019; Jornburom et al., 2020). Among various threats from humans, illegal hunting can severely reduce the chance of banteng recovery in forest habitat (Steinmetz, 2004). To control illegal hunting, the long‐term effective patrols in HKK have been one of the key management factors facilitating the recovery of bantengs in HKK. With further site security from patrol system in TYW, more secure areas have become available for bantengs to disperse beyond HKK, especially at the area across Mae Kong River next to the southern part of HKK.

We recommend the continuity of patrol quality to keep illegal hunting and other human threats at low level to allow more banteng to disperse and recover in TYW. The priority zone in TYW for habitat protection and management for banteng recovery is around low‐slope and dry dipterocarp forest areas adjacent to HKK. The monitoring system using camera traps should be continued and improved to study banteng habitat use patterns and provide further data to support banteng conservation in the TYW.

## AUTHOR CONTRIBUTIONS

**Peerawit Amorntiyangkul:** Investigation (equal); methodology (equal); project administration (equal); writing – original draft (equal). **Pornkamol Jornburom:** Formal analysis (supporting); funding acquisition (supporting); project administration (supporting); validation (supporting); writing – review and editing (supporting). **Anak Pattanavibool:** Data curation (supporting); methodology (supporting); writing – review and editing (supporting). **Warong Suksavate:** Formal analysis (supporting); methodology (supporting); supervision (supporting); validation (supporting); writing – review and editing (supporting). **Supalerk Klanprasert:** Investigation (supporting); project administration (supporting); resources (supporting); supervision (supporting). **Sahasawat Kaewvisat:** Data curation (supporting); investigation (supporting); project administration (supporting); resources (supporting); supervision (supporting). **Thanadid Thongthai:** Data curation (supporting); investigation (supporting); project administration (supporting); resources (supporting); supervision (supporting).

## FUNDING INFORMATION

The author(s) disclose receipt of the following financial support for the research, authorship, and/or publication of this article. This study was supported by US Fish and Wildlife Service, Liz Claiborne and Art Ortenberg Foundation, Arcadia Charitable Trust, Robertson Foundation, and the Wildlife Conservation Society Thailand Program.

## CONFLICT OF INTEREST STATEMENT

The authors have no conflicts of interest to declare.

## Data Availability

The presence/Absence of banteng in TYW, Thailand can be accessible on Dryad https://datadryad.org/stash/share/JSzS8HqXrjyxo_YSaceS2P8ghBd6FuAQlr825BBspA8.
